# Anatomy of Hematopoiesis and Local Microenvironments in the Bone Marrow. Where to?

**DOI:** 10.3389/fimmu.2021.768439

**Published:** 2021-11-11

**Authors:** Qingqing Wu, Jizhou Zhang, Daniel Lucas

**Affiliations:** ^1^ Division of Experimental Hematology and Cancer Biology, Cincinnati Children’s Medical Center, Cincinnati, OH, United States; ^2^ Department of Pediatrics, University of Cincinnati College of Medicine, Cincinnati, OH, United States

**Keywords:** anatomy of the bone marrow, spatial organization of hematopoiesis, microenvironment, niches, dynamics of hematopoiesis

## Abstract

The shape and spatial organization -the anatomy- of a tissue profoundly influences its function. Knowledge of the anatomical relationships between parent and daughter cells is necessary to understand differentiation and how the crosstalk between the different cells in the tissue leads to physiological maintenance and pathological perturbations. Blood cell production takes place in the bone marrow through the progressive differentiation of stem cells and progenitors. These are maintained and regulated by a heterogeneous microenvironment composed of stromal and hematopoietic cells. While hematopoiesis has been studied in extraordinary detail through functional and multiomics approaches, much less is known about the spatial organization of blood production and how local cues from the microenvironment influence this anatomy. Here, we discuss some of the studies that revealed a complex anatomy of hematopoiesis where discrete local microenvironments spatially organize and regulate specific subsets of hematopoietic stem cells and/or progenitors. We focus on the open questions in the field and discuss how new tools and technological advances are poised to transform our understanding of the anatomy of hematopoiesis.

## Introduction

The bone marrow tissue provides a unique microenvironment -composed of both hematopoietic and non-hematopoietic cells and extracellular matrix- that cooperate to accomplish several functions: promote stem cell and multipotent progenitor self-renewal, regulate the differentiation of each lineage, and provide structural support and spatial organization to the tissue. The microenvironment is defined by three large structures: the bone tissue that encloses the marrow; a vascular network, composed of arterioles that penetrate through the bone and give rise to a large sinusoidal network that drains through a central vein; and a network of reticular stromal cells that wraps around the different vessels. These structures cooperate with and regulate each other to maintain the tissue (1).

Many other cell types regulate -directly or indirectly- hematopoiesis and are thus considered part of the microenvironment. Non-hematopoietic cells include osteoblastic precursors, osteoblasts, osteocytes, adipocytes, Schwann cells, sympathetic and sensory nerves, and fibroblasts. Hematopoietic components include macrophages, megakaryocytes, myeloid cells, and dendritic cells. In-depth discussions on how these cells were recognized as components of the microenvironment and their precise role on hematopoiesis are available elsewhere (1–3). The components of the microenvironment are not evenly distributed through the bone marrow. As a result the microenvironment is spatially heterogeneous. Different regions of the bone marrow contain specialized microenvironments that organize hematopoiesis and regulate unique progenitors, cell types, and blood lineages. The next section discusses the evidence demonstrating that local microenvironments dictate the anatomy of hematopoiesis.

## The Anatomy of Hematopoiesis Is Spatially Organized by Local Microenvironments

### Spatial Organization of Hematopoietic Stem Cells and Their Niches

The discovery of the SLAM markers allowed imaging of HSC (defined as Lin^-^CD48^-^CD41^-^CD150^+^ cells) for the first time. This first study showed that most HSC were in perivascular location -in contrast to the established paradigm that stated that HSC were enriched in endosteal regions (4). It also paved the way for many other studies that used imaging to identify proximity between candidate niche cells and HSC and then a functional role for the niche cell was confirmed by genetic loss of function experiments (5–10). The composition, spatial organization, and function of HSC niches has been reviewed in detail elsewhere (1–3). Due to the sheer abundance of sinusoids and perivascular cells in the bone marrow virtually all (99%) hematopoietic cells –including HSC– localize within 30μm sinusoids or perivascular stromal cells (8, 11, 12). Both cell types are key regulators of HSC function (1, 3). Additionally, small subsets of HSC also localize near arterioles and/or the endosteum. Myeloid-biased HSC (detected using von Willebrand factor reporter mice) selectively localized near megakaryocytes -a key niche component that promotes HSC quiescence (9, 13, 14)- in the sinusoids. In contrast, lymphoid-biased HSC selectively localized near arterioles (15). Depletion of megakaryocytes led to expansion of myeloid biased HSC through loss of quiescence while lymphoid-biased HSC were unaffected. Similarly, depletion of Ng2^+^ periarteriolar stromal cells led to loss of the lymphoid-biased HSC (15). Other studies showed that the fraction of HSC with lowest levels of reactive oxygen species was enriched near arterioles (16); that increases in arteriole numbers also cause increases in HSC frequency (17); and that Ng2^+^ periarteriolar cells support HSC function (6). Together these results support the concept that sinusoids and megakaryocytes provide a niche for myeloid-biased LT-HSC whereas arterioles provide a niche for lymphoid-biased HSC. There is also evidence supporting the existence of an endosteal HSC niche that promotes regeneration. Imaging of fluorescently labeled HSC shortly after transplantation showed that the donor HSC are selectively enriched near the endosteal surface (18–20). Studies from the Li lab propose that CD49b^-^ cells represent a small subset of HSC that selectively amplifies in the endosteum -supported by N-cadherin^+^ stromal cells- in response to chemotherapy (21). In agreement, live imaging analyses showed that a rare HSC subset (MFG HSC) localized and amplified near the endosteum after chemotherapy treatment (22).

It is important to note that although most studies agree with the overall distribution described above there are ongoing controversies regarding whether some HSC selectively localize – and are maintained- by arteriolar and endosteal niches (8, 23); whether HSC localization to different niche components is selective or random [and thus controlled by the relative abundance of each niche component (12)]; and about the motility of HSC in live imaging analyses (22, 24). These are likely because each group has used different cell surface markers, transgenic reporters, and statistical approaches to identify HSC and niche cells and to test for spatial relationships between these cells.

### Spatial Organization of Hematopoietic Progenitors Downstream of HSC: Role of the Microenvironment

HSC give rise to several types of multipotent (MPP) and oligopotent progenitors (25–27). The localization of these cells in the microenvironment and whether they map near HSC and their niches is controversial. Early studies relied on short-term tracking of fluorescently-labeled MPP and HSC adoptively transplanted into non-myeloablated recipients. These revealed that the transplanted HSC and MPP did not overlap and that MPP localized further away from the endosteum than HSC (19). Much more recently, the Camargo lab generated Mds1^GFP+^ and Mds1^GFP+^Flt3-cre mice to differentially image subsets of multipotent progenitors and HSC. In the Mds1^GFP+^ mice GFP labels almost all HSC and subsets of MPP. In the Mds1^GFP+^Flt3-cre mice constitutive cre-mediated deletion of the floxed gfp allele restricts GFP expression to a small subset of HSC. They found that GFP^+^ cells in Mds1^GFP+^ mice were closer to transition zone vessels and farther away from the endosteum when compared to GFP+ cells in the Mds1^GFP+^Flt3-cre mice. This suggests that MPP and HSC reside in different microenvironments (22). The Pereira lab defined multipotent progenitors as Lin^-^CD41^-^CD48^-^cKIT^-^CD150^-^FLT3^+^ [which corresponds to the MPP4 subset (25)] and found a similar spatial distribution and interaction with perivascular stromal cells as HSC suggesting that they occupy the same niches (28). The differences between these studies are likely due to the different mouse reporters and methods used to image the multipotent progenitors.

It is likely that multipotent progenitors and lineage-committed progenitors do not overlap. *In vivo* imaging of adoptively transferred multipotent (Lineage^-^Sca1^+^c-kit^+^) or lineage-committed (Lineage^-^Sca1^-^c-kit^+^) progenitors into non-myeloablated recipients showed that both cells did not cluster and remained largely immobile while contacting the surrounding microenvironment (29). This suggested the existence of discrete niches for multipotent and lineage-committed progenitors. The existence of a distinct niche for erythropoiesis comes from classical electron microscopy studies that showed that rare macrophages, adjacent to sinusoids, provide a niche for islands of erythroblast maturation (30), and these have been the focus of many studies in the field (31). More recently, Comazzetto et al., demonstrated imaging of unipotent erythroid progenitors and showed that they selectively localize next to perivascular stromal cells that maintain them *via* SCF production (32). These indicate that erythropoiesis takes place in the sinusoids.

Herault et al., imaged Lineage^-^Sca1^-^CD150^-^c-kit^+^FcγR^+^ committed myeloid progenitors (33). These are a mixed population, containing granulocyte monocyte progenitors, and unipotent monocyte or neutrophil progenitors (34, 35). These myeloid progenitors were found as single cells evenly distributed through the bone marrow. In response to inflammation they formed large clusters that required signals provided by megakaryocytes to emerge (33). We recently developed strategies to image granulocyte progenitors, monocyte progenitors, monocyte dendritic cell progenitors (MDP) and most steps of terminal myeloid cell production (36). We found that myeloid progenitors do not colocalize with each other or HSC. Instead, they spatially segregate and attach to different sinusoids –away from arterioles and the endosteum– where they cluster with differentiated cells: granulocyte progenitors give rise and cluster with preneutrophils, monocyte progenitors cluster with Ly6C^hi^ monocytes, and MDP cluster with dendritic cells and Ly6C^lo^ non-classical monocytes. CSF1 is a key cytokine required for monocyte and dendritic cell production (37). When searching for microenvironmental signals that regulate this distribution we noticed that dendritic cells -which cluster with MDP- selectively localized to a rare subset of CSF1+ sinusoids (8% of all vessels). Conditional *Csf1* deletion in the vasculature led to loss of MDP, dendritic cells, and non-classical monocytes. The surviving MDP no longer attached to sinusoids nor formed clusters with dendritic cell or monocytes. These demonstrated that myelopoiesis is spatially organized by signals produced by discrete sinusoids and that CSF1^+^ sinusoids provide a unique microenvironment for dendritic cell production (36).

Several studies indicate that B cell differentiation is spatially organized and regulated by the microenvironment [for a recent review see (38)]. Common lymphoid progenitors distribute between the endosteum and arterioles and are maintained by CXCL12 produced by osteoblastic cells (targeted using *Col2.3-cre* or *Osx-cre* mice) and stem cell factor produced by osteolectin^+^ periarteriolar stromal cells (39–41). Cordeiro-Gomes found that Ly6D^+^ common lymphoid progenitors were also in contact with -and maintained by- a subset of IL7-producing perivascular stromal cells but it is not clear whether these stromal cells are evenly distributed through the bone marrow or enriched in specific locations (28). Interestingly, subsets of stromal cells predicted to support lymphopoiesis selectively localize near the growth plate and trabecular regions (42). The Nagasawa lab showed that most Pre-pro-B cells are in contact with CXCL12-producing stromal reticular stromal cells but did not localize near IL7-producing reticular cells. In contrast most Pro-B cells did not contact CXCL12 producing cells but localized near IL7 producing cells (43). Mandal et al., showed that Pre-B cells and Immature B cells selectively localize near IL-7^-^CXCL12^+^ reticular cells and that CXCR4 (the ligand for CXCL12) was necessary for Pre-B cell differentiation (44). Yu et al., demonstrated that deletion of IFG1 in Osterix+ progenitors using Osx-cre mice did not affect common lymphoid progenitors but led to arrest of B-cell development at the Pro-B stage (45). Fistonich et al., found that approximately 50% of ProB cells were in contact with IL7^+^CXCL12^+^ reticular cells that simultaneously contacted with a PreB cell. This suggested an overlapping niche for these two populations (46). Interestingly, live imaging showed that while ProB cells are largely static and remain attached to the CXCL12^+^ reticular cells whereas PreB cells migrate between different reticular cells (46). Together these studies strongly suggest that -as B cell progenitors differentiate- they migrate between subsets of stromal cells producing different amounts of IL7, CXCL12, or IGF1. In contrast, the Mancini lab found that most reticular cells coexpress IL7, CXCL12 and LepR, that ProB cells localize near LepR^+^, and that ~15% of HSC colocalize with Pro-B cells –much higher than predicted from random distributions. They also identified Nidogen-1 as niche derived factor regulating lymphopoiesis (47). Since HSC map near IL7 producing cells (28) these suggest that HSC and B cell lymphopoiesis share overlapping niches.

## Open Questions and Future Developments

**Figure 1:**
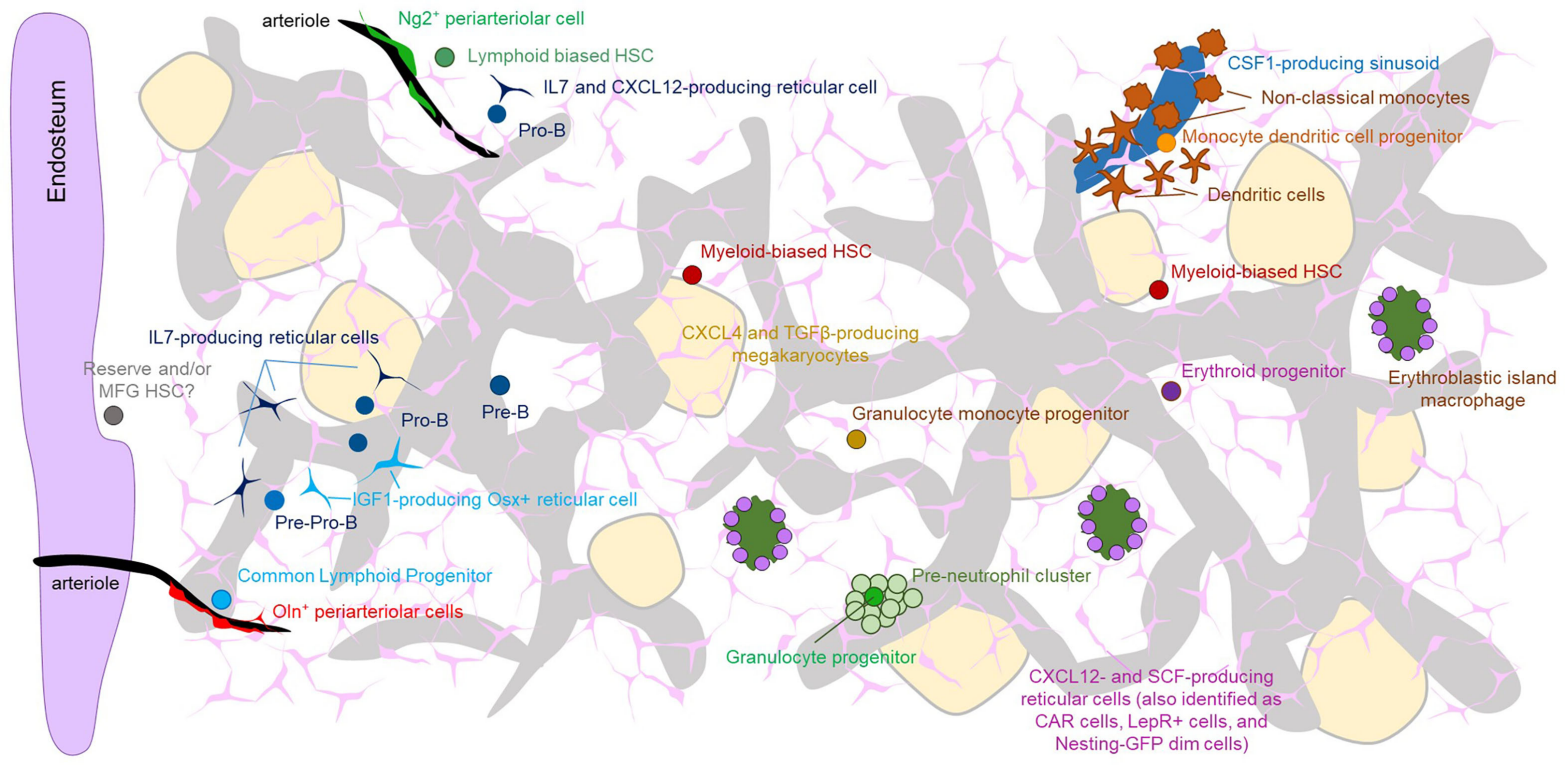
The Figure shows the overall architecture of the microenvironment in the bone marrow as well and the localization of the indicated progenitors with specific microenvironment. Note that because of the abundance of sinusoids and CXCL12- and SCF-producing perivascular cells virtually all cells are proximal to both of these structures. Also note that most types of stem cells and progenitors have been imaged with a limited number of partner cells. Therefore, it is likely that some of the structures depicted overlap (e.g. both erythroid progenitors and HSC have been shown to localize to SCF-producing perivascular cells). The precise location of most multi and oligopotent progenitors remains unknown.

The studies above demonstrate that the bone marrow is a complex organ with a unique spatial architecture where specific lineages are supported by discrete regions of the bone marrow (**Figure 1**). The studies also lead to new questions and reveal major gaps in our understanding of how spatial relationships regulate hematopoiesis.

### What Is the Anatomy of Stepwise Hematopoiesis in the Steady-State?

Hematopoiesis occurs *via* stepwise differentiation of progenitors. However, it has not been possible to map the location of many progenitor populations – including different subsets of multipotent progenitors, common myeloid progenitors, and megakaryocyte erythroid progenitors. Additionally, for most progenitors, it has not been possible to simultaneously image multiple types of progenitors. Therefore, it is not known whether different types of progenitors share the same niche (and are likely regulated by the same cells and structures) or different niches (which will suggest differential regulation). The main reasons limiting studies to answer these questions are technological. For example, progenitor populations can be routinely defined using complex multicolor flow cytometry panels (25–27). However, most confocal microscopes can only resolve a much more limited number of fluorescent channels. Additionally, scRNAseq studies demonstrated that many of the different flow gates used to prospectively isolate the different progenitors contain heterogeneous populations [e.g., heterogeneity of myeloid progenitors (34)]. Precise mapping of the different steps of blood maturation will require developing approaches to define each type of progenitor by using fewer fluorescence channels as done recently for stepwise mapping of myelopoiesis (36). Alternatively, it might be possible to adapt iterative imaging methods. In these, the samples are stained and imaged with a set of fluorescent probes followed by removal of the fluorescence and staining and imaging with new fluorescent probes. Two of these methods, CODEX and IBEX, are able to resolve dozens of parameters using confocal microscopy (48, 49).

Precise mapping of differentiation will also require clonal fate-mapping to determine developmental relationships between progenitors and adjacent cells. Different studies have used confetti mice [in which cre recombination leads to expression of one out of four fluorescent proteins (50) to examine clonal relationships between cells of interest in the marrow (22, 51)]. However, the confetti model only allows simultaneous detection of a very limited number of fluorescent tags in discrete progenitor populations. A possible way of overcoming this limitation is single-cell spatial transcriptomics, which is developing at a breakneck pace. It might soon be possible to obtain transcriptomic data, track thousands of barcodes for clonal analyses, and obtain spatial information for single cells in the bone marrow (52).

### What Are the Cells and Extracellular Matrix Structures Forming These Specialized Microenvironments and How Do They Function?

Answering these might require microdissection of the region of interest followed by transcriptomics analyses to interrogate the identity of the local cells. This technology is already available as shown by a study from the Van Galen lab demonstrating heterogeneity of growth factor production in different regions of the bone marrow (53).

After identification of the components of each local microenvironment the next step will be defining how they function in regulating the proximal progenitors. This has been accomplished by conditional Cre-mediated deletion of one cytokine or growth factor in the candidate cells. However, scRNAseq revealed extraordinary complexity of stromal cell types (53–55) whereas common Cre drivers available to the field target broad, heterogeneous, populations of stromal cells (55, 56). Development of new Cre^ERT2^ mouse models, specific for cells in local microenvironments -as done recently with *Oln-cre^ERT^* mice to target the periarteriolar stromal cells that maintain common lymphoid progenitors (41)- will greatly facilitate answering these questions.

### If Local Microenvironments Regulate Unique Stem/Progenitors What Regulates Progenitor Localization to These Structures?

One possibility is that the specialized microenvironment produces one or more chemotactic cues that selectively attract the desired progenitor. Alternatively, this process might be stochastic with progenitors migrating through the bone marrow transiently interacting with stromal components. This type of transient interactions was shown recently for HSC (24). Eventually, one of these interactions will be of sufficient strength and specificity to retain the progenitor in a specific microenvironment. In this case the relative abundance of each local microenvironment will profoundly influence the likelihood of successful interactions. A third possibility is that the stem/progenitors themselves remodel local cells into a supportive microenvironment. This type of remodeling has been shown to occur in the zebrafish HSC niche (57). Distinguishing between these possibilities will require live imaging of specific subsets of progenitors. The major technical constrain will likely be the development of fluorescent reporter strains to allow visualization of unique progenitor subsets. Live bone marrow imaging has been done in the mouse calvarium [where the bone is thin enough to allow imaging with sufficient resolution (19, 22)] or by carving a “window” in leg bones to image the marrow within (24, 29, 58). Importantly, recent studies have shown differences in the frequencies of erythroid and lymphoid progenitors across different bones (42, 59). These would have to be considered when deciding which bones to study *via* live imaging.

### What Is the Anatomy of Hematopoiesis and Local Microenvironment in Response to Insults?

Hematopoiesis is highly plastic and capable of sensing different insults and respond by quickly adjusting blood cell production to demand. Examples of this plasticity include hemorrhage which triggers emergency red blood cell production and infection which -depending on the infectious agent- can trigger emergency neutrophil, monocyte, and/or dendritic cell production. The bone marrow microenvironment plays critical roles in both sensing and orchestrating the progenitor response to infection [reviewed in (60)]. Importantly, inflammation and infection also profoundly remodel the sinusoidal network and perivascular stromal cells that maintain hematopoiesis and perturb stem cell localization within the marrow (60). Key open questions are a) whether hematopoietic stress responses use the same anatomical structures as normal hematopoiesis or instead depend on stress-specific anatomical cues and b) to what extend remodeling of sinusoids perturbs the anatomical structures that maintain the different progenitors. The most dramatic example of acute insult to the bone marrow is myeloablation. This eliminates not only hematopoietic cells but also the sinusoids and associated perivascular cells whereas endosteal regions and arterioles are more protected (6, 21, 61, 62). In this case the key open questions are: a) how are the local microenvironments restored? and b) what are the anatomical structures that support regenerative hematopoiesis? Identification of these will likely lead to novel therapies to promote restoration of blood cell production after myeloablation.

Chronic insults also lead to progressive remodeling of the microenvironment diminishing its capacity to support normal progenitors and -in some cases- hijacking it to promote pathogenesis. Examples of this pathogenic remodeling occur during physiological aging, leukemia, and other proliferative diseases (63, 64). Most studies have focused on determining how this remodeling perturbs HSC function. Little is known about how the different pathologies affect the structures that support more mature cells and whether protecting these structures can maintain normal hematopoiesis during disease.

## Conclusion

Hematopoiesis in the bone marrow is spatially and regionally organized by specialized local microenvironments that support different types of stem cells and progenitors. The challenges in imaging the bone marrow tissue have limited progress (65). However, the future is bright. Adapting technological advances validated in other tissues -including live imaging, multiparameter microscopy, new reporter strains, and spatial transcriptomics- will allow systematic examination of blood production *in situ* to define how local cues from the microenvironment control normal and pathological hematopoiesis.

## Author Contributions

DL conceived the manuscript who was written by all the authors. All authors contributed to the article and approved the submitted version.

## Funding

JZ is supported by an EvansMDS Young Investigator Award. DL is supported by R01 HL136529, R01 HL153229, R01 HL158616, and U54 DK126108.

## Conflict of Interest

The authors declare that the research was conducted in the absence of any commercial or financial relationships that could be construed as a potential conflict of interest.

## Publisher’s Note

All claims expressed in this article are solely those of the authors and do not necessarily represent those of their affiliated organizations, or those of the publisher, the editors and the reviewers. Any product that may be evaluated in this article, or claim that may be made by its manufacturer, is not guaranteed or endorsed by the publisher.
